# Systematic review and meta-analysis of the efficacy of prophylactic abdominal drainage in major liver resections

**DOI:** 10.1038/s41598-021-82333-x

**Published:** 2021-02-04

**Authors:** Sepehr Abbasi Dezfouli, Umut Kaan Ünal, Omid Ghamarnejad, Elias Khajeh, Sadeq Ali-Hasan-Al-Saegh, Ali Ramouz, Roozbeh Salehpour, Mohammad Golriz, De-Hua Chang, Markus Mieth, Katrin Hoffmann, Pascal Probst, Arianeb Mehrabi

**Affiliations:** 1 Head of the Division of Liver Surgery and Visceral Transplantation, Department of General, Visceral, and Transplantation Surgery, University of Heidelberg, Im Neuenheimer Feld 420, 69120 Heidelberg, Germany; 2 Department of Diagnostic and Interventional Radiology, University of Heidelberg, Heidelberg, Germany; 3 Liver Cancer Center Heidelberg (LCCH), Heidelberg, Germany

**Keywords:** Cancer, Cancer, Surgical oncology

## Abstract

Prophylactic drainage after major liver resection remains controversial. This systematic review and meta-analysis evaluate the value of prophylactic drainage after major liver resection. PubMed, Web of Science, and Cochrane Central were searched. Postoperative bile leak, bleeding, interventional drainage, wound infection, total complications, and length of hospital stay were the outcomes of interest. Dichotomous outcomes were presented as odds ratios (OR) and for continuous outcomes, weighted mean differences (MDs) were computed by the inverse variance method. Summary effect measures are presented together with their corresponding 95% confidence intervals (CI). The certainty of evidence was evaluated using the Grades of Research, Assessment, Development and Evaluation (GRADE) approach, which was mostly moderate for evaluated outcomes. Three randomized controlled trials and five non-randomized trials including 5,050 patients were included. Bile leakage rate was higher in the drain group (OR: 2.32; 95% CI 1.18–4.55; p = 0.01) and interventional drains were inserted more frequently in this group (OR: 1.53; 95% CI 1.11–2.10; p = 0.009). Total complications were higher (OR: 1.71; 95% CI 1.45–2.03; p < 0.001) and length of hospital stay was longer (MD: 1.01 days; 95% CI 0.47–1.56 days; p < 0.001) in the drain group. The use of prophylactic drainage showed no beneficial effects after major liver resection; however, the definitions and classifications used to report on postoperative complications and surgical complexity are heterogeneous among the published studies. Further well-designed RCTs with large sample sizes are required to conclusively determine the effects of drainage after major liver resection.

## Introduction

Major liver resection is considered the treatment of choice in patients with large and multiple lesions^1^. Procedure-related complications can occur following major hepatectomy, and despite remarkable improvements in the surgical technique, morbidity rates remain high^2^. Prophylactic drain insertion has been a routine practice in abdominal surgery. These drains are inserted to detect and characterize bleeding or abdominal collections early on and to prevent and manage postoperative fluid collection^3^. However, in recent years, this widespread practice has been abandoned as a routine part of many operations, such as cholecystectomy^4^, pancreatic surgery^5^, and standard bowel resections^6^. Innovations in imaging techniques and advancements in various diagnostic tests have made it possible to identify fluid collections without a prophylactic drain. When necessary, these complications can be managed with interventional percutaneous drainage.

Whether prophylactic drains are necessary in liver resection has been discussed in recent studies. As a matter of fact, recent randomized controlled trials (RCTs) showed the validity of hepatectomy without abdominal drainage^7–9^. Some showed that prophylactic drains were linked with higher leaks, prolonged length of hospital stay, and increased costs^10–12^. However, these studies included mostly minor liver resections. Since major liver resections are associated with a significantly higher rate of morbidity and mortality^7–10^, the role of abdominal drain insertion after major liver resection still remains controversial among liver surgeons. To the best of our knowledge, there is no meta-analysis comparing the results exclusively after major liver resections in patients with intraoperative abdominal drains and those without. Furthermore, existing RCTs have compared the results after all types of liver resection (including minor and major resections) so we do not know how drainage influences the outcome of major resection specifically. Because of this lack of reliable evidence, there are no concrete recommendations concerning the use of prophylactic drainage in major liver resections. Nevertheless, prophylactic drains are being routinely placed after major liver resections in many hospitals.

This systematic review and meta-analysis was conducted to determine the value of prophylactic drainage after major liver resection.

## Results

**Figure 1:**
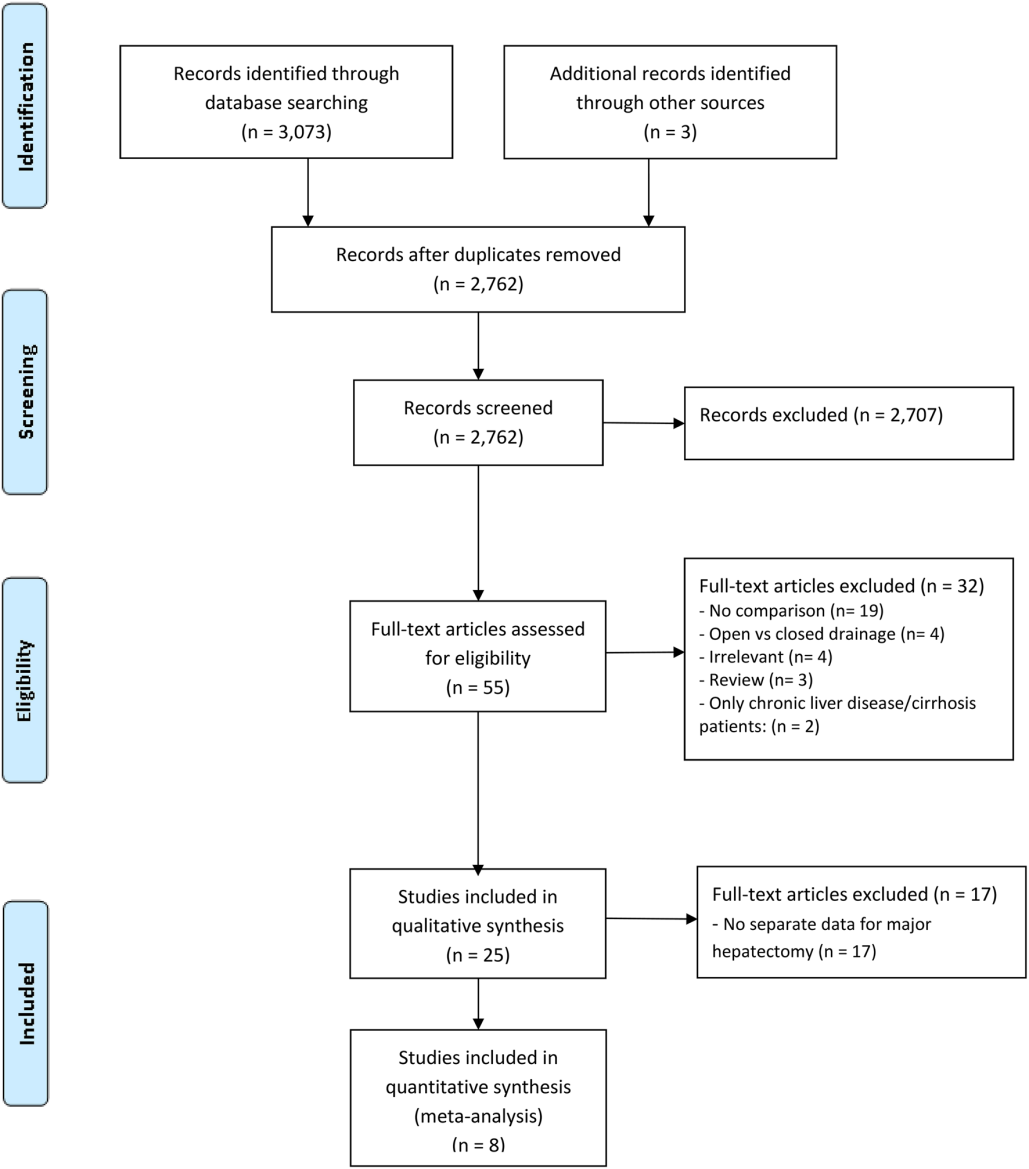
PRISMA flow chart of study selection.

The systematic searches yielded 3,146 articles. Title and abstract screening excluded 2765 publications that met the predetermined exclusion criteria (Fig. 1). After full-text evaluation of the remaining 58 articles, three RCTs and five non-randomized studies were considered eligible. From these 58 articles, 18 (4 RCTs and 14 non-randomized studies) were excluded because data on major liver resections could not be extracted. The included studies contained 5,050 patients who underwent major liver resection.

### Risk of bias assessment for included studies

From the eight included articles, three were RCTs including a total of 241 patients (122 with drainage and 119 without)^8,13,14^, and five were retrospective studies including 4809 patients (2548 with drainage and 2,261 without)^15–19^. All were published between 1989 and 2018 (Table 1).

**Table 1:** Details and characteristics of studies.

Author year	Country	Study type	Number of patients with liver resection	Number of patients with major liver resection			Inclusion criteria	Exclusion criteria	Type of drain	Indications for surgery	Cirrhotic patients
				Total	Drain used	No drain used					
Belgithi 1993	France	RCT	81	24	14	10	NCD	Patients with: 1-Bilioenteric anastomosis (n = 6), 2-Gastrointestinal procedure (n = 6), 3-Extended hepatic resection with total vascular exclusion and/or ex situ perfused liver (n = 5), 4-Devitalized hepatic stump tissues (n = 2), 5-Injury to the common bile duct (n = 2), 6-Refusal to participate (n = 3)	Closed suction drain	42 (51.8%) with benign lesions and 39 (48.2%) with malignant tumors such as HCC (21 patients), CCC (4 patients) and metastasis (14 patients)	19 (23.4%)
Fong 1996	USA	RCT	120	87	44	43	NCD	Patients with: 1-Refusal to participate, 2-Thoracoabdominal biliary-enteric simultaneous anastomosis, 3-Preoperative placement of a biliary stent	Closed suction drain	84 (70%) had metastatic cancer and 36 (30%) had primary liver pathology	6 (5%) [2 in no-drain group and 4 in drain group]
Kim 2014	Korea	RCT	200	130	64	66	NCD	Patients with: 1-Surgeons preference (n = 1) 2-Intraoperative injury of hepatic duct (n = 1)	Closed suction drain	50 (25%) with benign lesions [21 in no-drain group and 29 in drain group] and 150 (75%) with malignant tumors [79 in no-drain group and 71in drain group]	108 (54%) [55 in no-drain group and 53 in drain group]
Aldameh 2005	New Zealand	NRS	211	111	79	32	Patients with resection of hepatic parenchyma only	Patients with: 1-Bilioenteric anastomosis	18F closed non-suction drain	31 (14.6%) with benign lesions [19 in no-drain group and 12 in drain group] and 146 (69.2%) with malignant tumors [54 in no-drain group and 92 in drain group] HCC [20 (9.5%)] Metastasis [109 (51.6%)] CCC [8 (3.8%)] Gallbladder cancer [9 (4.2%)]	NCD
Squires 2015	USA	NRS	1041	1041	564	477	NCD	Patients with: 1-Concurrent biliary reconstruction and anastomosis (n = 198)	Closed drain	215 (20.6%) with benign lesions [96 in no-drain group and 119 in drain group] and 822 (78.9%) with malignant tumors [381 in no-drain group and 441 in drain group] HCC [123 (11.8%)] Metastasis [626 (60.1%)] CCC [73 (7%)]	20 (2%) [7 in no-drain group and 13 in drain group]
Cauchy 2016	France	NRS	223	223	63	160	1-Patients undergoing full laparoscopic major right or left liver resection, 2-Lesions well clear of the midplane	Patients with: 1-Planned ‘‘hand-assisted’’ or ‘‘hybrid’’ approach, 2-Total vascular exclusion without or with liver cooling, 3-Reconstruction of major vascular were required	NCD	26 (11.7%) with benign lesions and 197 (88.3%) with malignant tumors HCC [44 (19.7%)] Metastasis [112 (50.2%)] CCC [27 (12.1%)] Others [14 (6.3%)]	NCD
Shwaartz 2017	USA	NRS	1005	1005	500	505	Patients with major liver resection	Patients with: 1. Minor hepatectomy (n = 1,246), 2. Biliary reconstruction, 3. Missing info (n = 17)	NCD	159 (15.8%) with benign lesions [71 in no-drain group and 88 in drain group] and 785 (78.1%) with malignant tumors [377 in no-drain group and 408 in drain group] Hepatobiliary cancers [271 (27%)] Metastasis [514 (51.1%)]	83 (8.2%) [28 in no-drain group and 55 in drain group]
Martin 2018	USA	NRS	6861	2429	1342	1087	Adult patients ≥ 18 years old	Patients with: 1.Missing bile leak variable (n = 59), 2.Unknown or missing final pathologic diagnosis (n = 7), 3.An ICD-9 code indicating neoplasm of uncertain behavior	NCD	5226 (76.2%) malignant tumors	NCD
Total			9742	5050	2670	2380					

*RCT* Randomized controlled trial, *NRS* non-randomized studies, *NCD* not clearly defined, *HCC* hepatocellular carcinoma, *CCC* cholangiocarcinoma.

As shown in Table 2, all three RCTs had some concerns of bias in the evaluated domains, including the randomization process, deviations from intended interventions, missing outcome data, measurement of the outcome, and selection of the reported result. Therefore, overall assessment of included RCTs showed some concerns regarding the risk of bias. Among non-randomized studies, all authors had clearly defined the aim of the studies, however, only the study by Squires et al.^19^ had included consecutive patients (Table 3). Although four studies benefited prospective data collection, Martin et al.^17^ reported no data regarding the data collection. The endpoints were defined appropriately in three studies, and the endpoint definition of two studies was not adequately reported. Only Shwaartz et al.^18^ reported the unbiased assessment of the endpoints but inadequately, whereas other studies reported no data in this regard. Data regarding the follow-up period was adequate only in study by Cauchy et al.^16^ and three studies reported the follow-up inadequately. Nonetheless, Shwaartz et al.^18^ did not report the data regarding the follow-up. Albeit inadequately, Martin et al.^17^ was the single study, which provided data of the patients lost to follow-up. Despite the study by Martin et al.^17^, all other non-randomized studies reported the prospective calculation of the sample size. Overall, four out of five non-randomized studies had intermediate quality^15,16,18,19^, and the remaining study^17^ presented poor quality (Table 3). A publication bias analysis was not performed because fewer than ten studies were included. The certainty of evidence for the outcomes, assessed using the GRADE approach, was moderate for majority of the considered outcomes.

**Table 2:** Risk of bias assessment for included randomized controlled trials.

Cochrane tool for assessing risk of bias for randomized trials (Cochrane RoB 2.0)			
First author	Kim	Fong	Belghiti
Q1 (Bias arising from the randomization process)	Some concerns	Some concerns	Some concerns
Q2 (Bias due to deviations from intended interventions)	Some concerns	Some concerns	Some concerns
Q3 (Bias due to missing outcome data)	Some concerns	Some concerns	Some concerns
Q4 (Bias in measurement of the outcome)	Some concerns	Some concerns	Some concerns
Q5 (Bias in selection of the reported result)	Some concerns	Some concerns	Some concerns
Total	Some concerns	Some concerns	Some concerns

*Q1* Was allocation sequence concealed? *Q2* Participant and personal aware of intervention? *Q3* Were outcome data available for all, or nearly all, participants randomized? *Q4* Were outcomes assessors aware of the intervention received by study participants? *Q5* reported data selected, on the basis of the results, from multiple outcomes or multiple analyses of the data?

**Table 3:** Risk of bias assessment for included non-randomized studies.

Methodological index for non-randomized studies									
Studies (first author)	Q 1	Q 2	Q 3	Q4	Q 5	Q6	Q 7	Q 8	Total
Aldameh et al	2	0	2	1	0	1	0	2	8 (intermediate)
Cauchy et al	2	0	2	1	0	2	0	2	9 (intermediate)
Shwaartz et al	2	0	2	2	1	0	0	2	9 (intermediate)
Squires et al	2	2	2	2	0	1	0	2	11 (intermediate)
Martin et al	2	0	0	2	0	1	1	0	6 (low)

*Q1* Did the study have a clear aim? *Q2* Were consecutive patients included? *Q3* Were data collected prospectively? *Q4* Were endpoints appropriate to the study? *Q5* Was there an unbiased assessment of endpoints? *Q6* Was the follow-up period adequate? *Q7* Was loss to follow-up less than 5%? *Q8* Was there a prospective calculation of study size? 0, Not reported; 1, reported but inadequate; 2, reported and adequate. Overall score rating: > 12, high; 8–12, intermediate; < 8, low.

### Definition of major hepatectomy

All included studies defined major hepatectomy as resection of three or more liver segments, except the study of Aldameh et al.^15^, which defined major hepatectomy as resection of four or more hepatic segments.

Central hepatectomy was only mentioned in the study of Squires et al.^19^, which included 17 patients in the non-drain group and seven patients in the drain group. The complications were not separately mentioned in these subgroups.

Bile duct reconstructions were performed in 3.6% of patients in the study of Cauchy et al.^16^ and in 1.3% of patients in the study of Martin et al.^17^. Both studies reported bile duct reconstruction as an independent risk factor for bile leak in their analysis, but they did not report the distribution of bile duct reconstruction in the two drain and no-drain groups.

### Type of intraoperative drains and time of removal

The type of drain used was not provided in three studies^16–18^. Squires et al.^19^ used closed drains; however, the suction type was not described. Belghiti et al.^13^, Fong et al.^14^, and Kim et al.^8^ reported using closed suction drains, whereas Aldameh et al.^15^ reported using closed non-suction drains.

Regarding the time of drain removal, Fong et al.^14^ removed drains after the fourth postoperative day if no bilious discharge was noted in the drainage, regardless of amount of drainage. Belghiti et al.^13^ removed the drains when the daily drainage was less than 100 mL, usually three to five days after the operation. A standardized timing for the removal of used drains was not clearly specified in the other included studies.

Because the type of drain used was not specified in some of the included studies, and because the time of drain removal was heterogeneous among the included studies, a subgroup analysis based on drain management was not feasible.

### Postoperative bile leak

There was considerable heterogeneity in the definition of bile leakage between the studies. Three authors used the ISGLS definition of bile leakage^16–18^. Fong et al.^14^ defined bile leakage as any volume of ongoing bilious drainage for more than one week postoperatively. Aldameh et al.^15^ defined a bile leakage as drainage of more than 50 mL of bile from the abdominal cavity per day for more than three days. Bile leakage was not defined in the remaining studies^8,13,19^.

**Figure 2:**
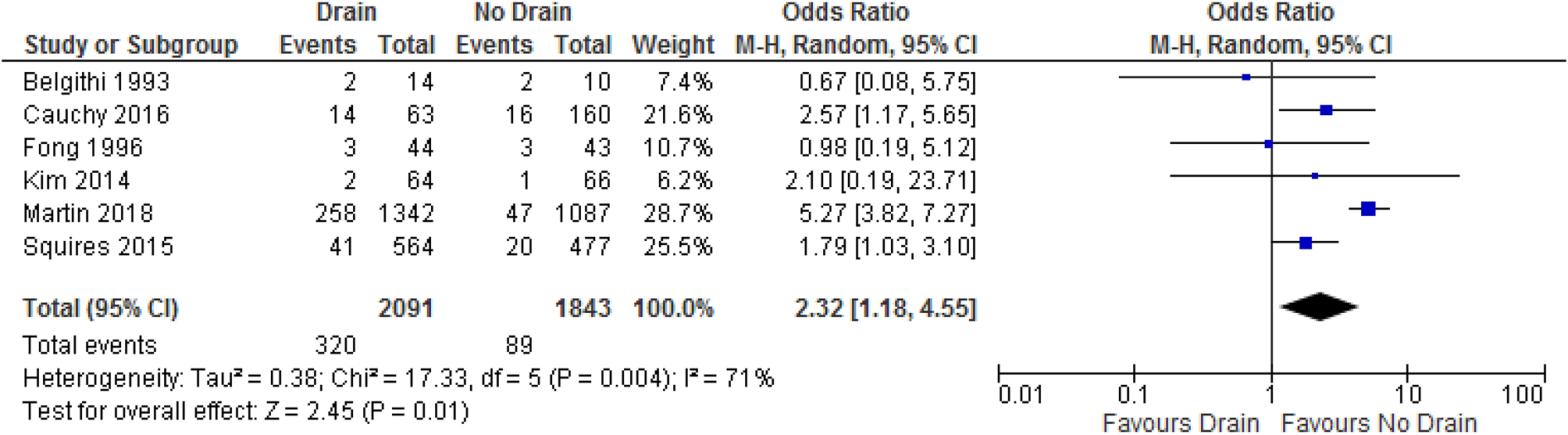
Forest plot of postoperative bile leak.

Six studies, including three RCTs and three non-randomized studies^8,13,14,16,17,19^, were included in the analysis of postoperative bile leakage. The study of Shwaartz et al.^18^ was excluded because it duplicated data from the study of Martin et al.^17^. The rate of bile leakage in the drain and no-drain groups were 15.3% and 4.8%, respectively. A pooled analysis of 4,939 patients demonstrated that drainage was associated with significantly higher incidence of bile leakage than no drainage was (OR: 2.32; 95% CI 1.18–4.55; p = 0.01; Fig. 2). There was significant heterogeneity between studies (I^2^ = 71%; p = 0.004) and the certainty of evidence was moderate. A separate subgroup analysis of the three RCTs did not confirm these results (OR: 1.05; 95% CI 0.34–3.29; p = 0.93; I^2^ = 0%; Supplemental Fig. 1) whereas a subgroup analysis of the non-randomized studies did (OR: 2.99; 95% CI 1.40–6.38; p = 0.005; I^2^ = 84%; Supplemental Fig. 2).

### Postoperative bleeding

Two RCTs evaluating 154 patients reported that five patients had postoperative bleeding or hematoma^8,13^. In the study of Belghiti et al. (N = 24)^13^, one case of bleeding and two intraabdominal hematomas were reported. The bleeding happened on the ninth day after a right hemihepatectomy in the non-drain group and was caused by a pseudoaneurysm on the stump of the right hepatic artery. This was successfully treated with a reoperation. Both hematomas were reported in the drain group. They were detected through routine postoperative ultrasound and were managed with percutaneous drains. The other two bleeding events were reported by Kim et al. (N = 130)^8^. Both bleedings occurred at drain sites and were sutured under general anesthesia.

**Figure 3:**
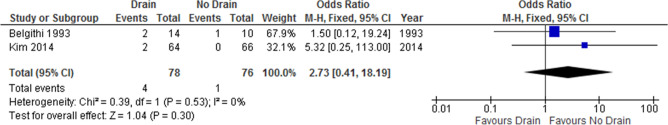
Forest plot of postoperative bleeding.

Two RCTs were included in the analysis of postoperative bleedings. The rate of postoperative bleedings in the drain and no-drain groups were 5.1% and 1.3%, respectively; however, a pooled analysis of 154 patients demonstrated that this difference was not significant (OR: 2.73; 95% CI 0.41–18.19; p = 0.30; Fig. 3). There was no considerable heterogeneity between studies (I^2^ = 0%, p = 0.53) and the certainty of evidence was high.

### Postoperative interventional percutaneous drain

**Figure 4:**
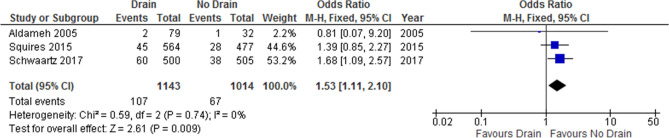
Forest plot of postoperative interventional percutaneous drain.

Interventional drains were fitted postoperatively after detecting a symptomatic fluid collection. In most studies, the indication for inserting a percutaneous drain was not defined (e.g., bile leakage or any symptomatic fluid collection). Three non-randomized studies^15,18,19^ were included in the analysis and the necessity of postoperative percutaneous drains in the drain and no-drain groups were 9.3% and 6.6%, respectively. A pooled analysis of 2157 patients demonstrated that drainage was associated with a significantly higher need for postoperative percutaneous drains than no drainage was (OR: 1.53; 95% CI 1.11–2.10; p = 0.009; Fig. 4). There was no considerable heterogeneity between studies (I^2^ = 0%, p = 0.74) and the certainty of evidence was low.

### Wound infection

**Figure 5:**
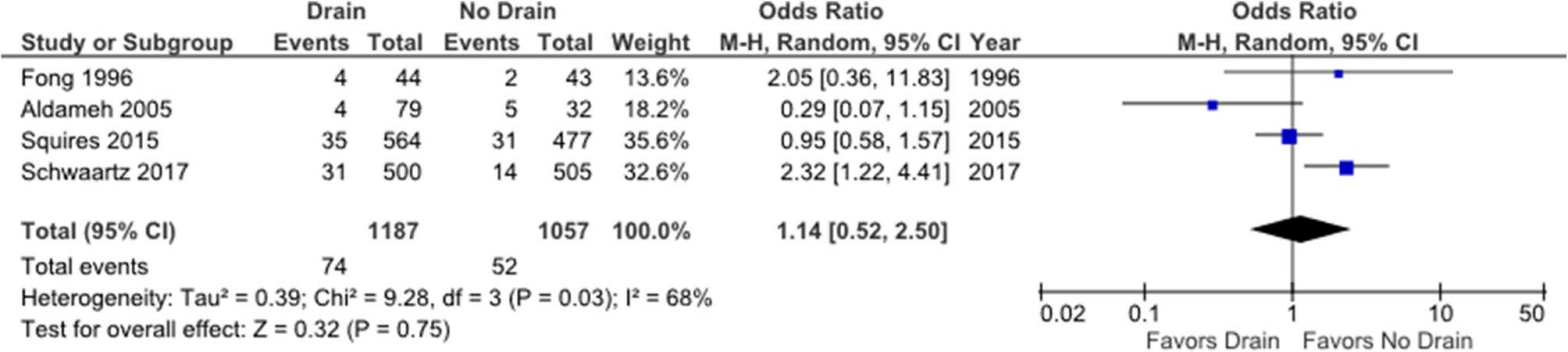
Forest plot of wound infection.

Four studies (one RCT^14^ and three non-randomized studies^15,18,19^) were included in the analysis of wound or drain site infection. The rates of wound infection in the drain and no-drain groups were 6.2% and 4.9%, respectively; however, a pooled analysis of 2244 patients demonstrated that this difference was not statistically significant (OR: 1.14; 95% CI 0.52–2.50; p = 0.32, Fig. 5). There was moderate heterogeneity between studies (I^2^ = 68%, p = 0.03) and the certainty of evidence was moderate.

### Total complications

**Figure 6:**
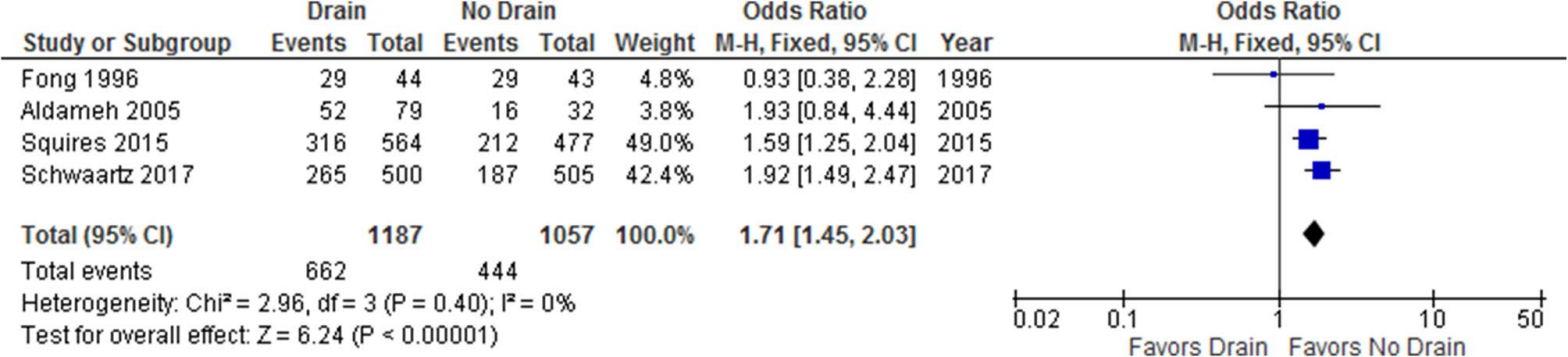
Forest plot of total complications.

Martin et al.^17^ and Shwaartz et al.^18^ reported the 30 postoperative day morbidity based on the NSQIP database. Squires et al.^19^ used the Clavien–Dindo scoring system^20^ to grade postoperative complications until the thirtieth postoperative day. Cauchy et al.^16^ also used the Clavien–Dindo system to report complications until the 90th postoperative day. Complications were not defined in the other studies. Four studies (one RCT^14^ and three non-randomized studies^15,18,19^) were included in the analysis of post-hepatectomy complications. The rate of post-hepatectomy complications in the drain and no-drain groups were 55.8% and 42.0%, respectively. A pooled analysis of 2244 patients demonstrated that drainage was associated with a significantly higher rate of post-hepatectomy complications than no drainage was (OR: 1.71; 95% CI 1.45–2.03; p < 0.001; Fig. 6). There was no considerable heterogeneity between studies (I^2^: 0%, p = 0.40) and the certainty of evidence was moderate.

### Length of hospital stay

**Figure 7:**
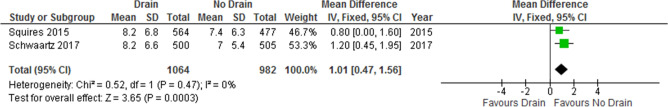
Forest plot of length of hospital stay.

Two non-randomized studies^18,19^ were included in the analysis of the length of hospital stay. A pooled analysis of 2,046 patients revealed a significantly longer hospital stay in the drain group than in the no-drain group (MD: 1.01 days; 95% CI 0.47 days to 1.56 days; p < 0.001, Fig. 7). There was no considerable heterogeneity between studies (I^2^: 0%, p = 0.47) and the certainty of evidence was moderate.

### Subgroup analysis of laparoscopic resections

The three oldest studies^13–15^ included only open resections. The study of Cauchy et al.^16^ included only laparoscopic major hepatectomies. The remaining studies included both open and laparoscopic hepatectomies, but these data were not reported separately. Therefore, the only data that could be used in a subgroup analysis for laparoscopic resections was from the study of Cauchy et al.^16^. This study reported only bile leak. The subgroup analysis showed similar results, with bile leak being significantly higher in the drain group than in the non-drain group (0.22% versus 0.1%; OR: 2.57; 95% CI 1.17–5.67; p = 0.02).

## Discussion

The main finding of this systemic review and meta-analysis was that prophylactic drainage did not improve the outcomes after major liver resection. In fact, prophylactic drainage even worsened some outcomes in retrospective studies, including a higher rate of bile leakage, more frequent insertion of postoperative interventional percutaneous drains, higher rate of total complications, and longer hospital stay. Minor liver resection has been shown to be safe without drainage^21,22^; therefore, many surgeons still tend to use routine intraoperative drains after major liver resections but not after minor resections. The reason is that postoperative complications are significantly higher after major resections. However, whether drainage reduces this complication rate is not known—this fact necessitated the current meta-analysis. Our study showed that the drains did not reduce bile leakage/bleeding or the need to insert secondary interventional drains. It seems that prophylactic drains fail to evacuate these collections in many cases. Possible reasons include secondary dislocation of the drains, septation of the abdominal collections, and development of abdominal collections after drain removal. Consequently, these patients have to go through the same diagnostic and therapeutic procedures as patients without drains.

Operative site drains have been routinely inserted during liver surgery for many decades. Whether or not these drains are beneficial after liver resection has been challenged in the past years, and some RCTs have suggested that drains are unnecessary^7–9^. Three meta-analyses have compared postoperative complications after liver resections^21–23^. Only Gurusamy et al. performed a subgroup analysis for major resections in 2007, but the number of patients extracted was small (N = 112). This analysis showed no difference in any of the outcomes except hospital stay, which was lower in the drain group; however, this difference was not significant after the random-effects model was applied. The meta-analyses showed that routine drainage does not reduce postoperative complications after liver resections. However, the majority of the operations were minor resections, and many questions remained unanswered concerning major liver resections. Major liver resections have significantly more complications than less extensive resections^24^, so the results of the aforementioned meta-analyses may not be applicable. Since the study of Gurusamy et al.^22^ in 2007, several articles with larger samples have reported and compared their outcomes of major resections, making the present up-to-date meta-analysis possible.

We included five non-randomized studies (N = 4,809) and three RCTs (N = 241) in this meta-analysis. Overall, the analysis favored the no-drain group, with some complications occurring significantly more often in the drain group. The meta-analysis for postoperative bile leakage showed a two-fold higher rate in the drain group, but this difference was not significant according to subgroup analysis of RCTs. The other complications were mentioned in only one RCT^14^, so a subgroup analysis for RCTs was not performed.

Four RCTs were excluded because they did not report data on major resections. Of these excluded studies, Sun et al.^9^ and Liu et al.^25^ found that wound complication and morbidity rate were higher in the drain group. Fuster et al.^26^ included only cirrhotic patients and observed a decreased ascites leakage and a reduced hospital stay in the drain group. The most recent study of Arita et al.^7^, which was a high volume multicentric study on 400 patients, showed that drain placement increases the rate of severe postoperative complications in patients undergoing hepatic resection, but only 12% of these resections were major and patients with a high intraoperative risk of bile leakage or hemorrhage were excluded from this study^7^.

The present study has shown that postoperative bleeding and hematomas are rare after major hepatectomy and do not justify placing a drain. In all three reported cases, drains did not help diagnose postoperative bleeding earlier and did not improve treatment. Drains even caused bleeding in two patients. Postoperative interventional percutaneous drains were inserted in two-fold more patients in the drain group than in the no-drain group. Wound complication rates were the same in both groups, but postoperative total morbidity was higher and hospital stay was longer in the drain group.

Only well-designed RCTs with adequate sample sizes can provide reliable answers. Because the number of patients extracted from RCTs was low, most studies included in this analysis were retrospective. Hence the above-mentioned findings should be interpreted with caution because the risk of bias is high. Indications for inserting intraoperative drains were different between the studies. The decision to insert a drain was mostly made by the surgeons and it seemed that in some retrospective studies, drains were inserted because of concerns about bile leakage or bleeding. As a result, intraoperative drains were mostly inserted after more complicated resections and this could explain the higher rate of postoperative complications in the drain group. In most studies, it was not mentioned if central liver resection or bile duct reconstruction was performed. These two surgical methods are related to higher bile leak rates^17^, so homogeneity between the groups cannot be evaluated. The OR of bile leakage was 1.04 in RCTs and 2.99 in retrospective studies, indicating that the drain was inserted in high-risk patients for bile leakage. On the other hand, reported complications were only ISGLS grade B and C in the no-drain group^27^, whereas grade A, B, and C bile leaks were reported the drain group. This suggests that inserting a drain may lead to over diagnosis of a non-complicated bile leakage (grade A), fluid collection, or ascending infections^28,29^, which may in turn lead to further therapeutic/diagnostic interventions. This might also explain the higher overall morbidity, longer hospital stay in the drain group, which leads to higher costs. Furthermore, drains may also increase patient discomfort^17,30,31^.

The use of laparoscopic liver resection is increasing worldwide, so we performed a subgroup analysis for laparoscopic resections. This analysis showed the same results, indicating that bile leakage is independent of whether the resection is open or laparoscopic.

There are some limitations to the present meta-analysis. No RCT exclusively compared complications between drain and no-drain groups following major liver resection. Only three out of seven existing RCTs (with a total sample size of 241 patients) reported major liver resection as a subgroup. The included studies were also published over a large time frame (Belgithi et al.^13^ and Martin et al.^17^), so improvements in liver surgery made during this time could potentially influence the results. In addition, there is considerable heterogeneity between the studies, including indications and type of inserted intraoperative drains, definitions of bile leakage, indications for inserting secondary percutaneous drains, time of drain removal, and type of major liver resection (including central liver resection and performance of bile duct reconstruction). Because of this heterogeneity, further subgroup analyses (based on the type of drains, type of resection, bile duct reconstruction, etc.) were not possible. Because of this heterogeneity together with the small sample size extracted from RCTs, the recommendation derived from our meta-analysis cannot be very strong. Furthermore, the indications for liver resection are constantly widening. The increase in neoadjuvant chemotherapy and the growing complexity of surgical procedures may also influence postoperative complications. This imposes a cautious attitude in these patients. Hence further well-designed, large-scale RCTs with standardized definitions of the above-mentioned factors are needed to accurately compare complications following major liver resection with and without drainage.

## Methods

The present study was reported according to Preferred Reporting Items for Systematic Reviews and Meta-Analyses (PRISMA) guidelines^32^.

### Eligibility criteria

The research question and eligibility criteria were formulated based on the PICOS strategy (population, intervention, comparison, outcomes, and design of studies).

- *Population:* all adult cases who underwent major hepatectomy
- *Intervention:* prophylactic intraoperative abdominal drainage
- *Comparators:* no prophylactic intraoperative abdominal drainage
- *Outcome:* postoperative bile leak, postoperative bleeding, postoperative interventional percutaneous drain, wound infection, total complications, length of hospital stay
- *Study design:* any study design except case reports, study protocols, animal studies, conference papers, and letters to the editor.

To eliminate the risk of analyzing the same patients more than once, the studies were thoroughly assessed and double publications and overlapping reports were removed. The remaining studies were selected for full-text review by reviewing the titles and abstracts for eligibility criteria.

### Literature search

The predefined search terms were: (“opened suction" OR "open suction" OR "open conduit" OR "drainage" OR "drain*" OR "easy flow" OR "closed suction" OR "close suction") AND ("liver" OR "hepatic") AND ("resection" OR "hepatectomy"). Our comprehensive literature search was conducted to identify relevant articles in the Medline/PubMed, Web of Science and Cochrane Central databases from their inception to October 2020. A recent publication showed that PubMed/Medline and Cochrane Central need to be searched for systematic reviews of RCTs in the field of surgical interventions as a minimum^33^. For systematic reviews including non-randomized studies, Web of Science should be added. EMBASE does not contribute substantially to reviews on surgical interventions^33^. All studies comparing postoperative outcomes in adult patients who underwent major hepatectomy with and without prophylactic abdominal drainage were included.

### Study selection

Three authors (UKÜ, SAS, and RS) independently screened all titles and abstracts and made their selections according to PICOS eligibility criteria. The full-text of appropriate studies were evaluated and their data were extracted by three authors (UKÜ, OG, and EK) independently. To assure that the extracted data reflect only major hepatectomies we only included studies, which reported the complications after major hepatectomies separately and studies with mixed data were excluded. Discrepancies among these investigators were resolved through discussions with a senior author (AM). For each treatment group, the following data were extracted: study characteristics, patient characteristics, study quality, and the outcome measures described above.

### Critical appraisal

Two investigators (SAD and MG) assessed the bias of each study independently using the methodological index for non‐randomized studies (MINORS)^34^ or the Cochrane RoB 2.0^35^. Items in the MINORS index were scored as 0 (not reported), 1 (reported but inadequate), and 2 (reported and adequate). Non-randomized studies with less than eight points were considered poor quality, 8–12 points intermediate quality, and more than 12 points high quality. The methodological quality of RCTs was assessed by Cochrane RoB 2.0^35^. The five domains of Cochrane RoB 2.0 included bias due to (1) the process of randomization, (2) deviations from intended interventions, (3) missing outcome data, (4) measurement of the outcome, and (5) selection of the reported results. Items were judged as low risk of bias, some concern of bias, or high risk of bias. To determine the overall quality of outcome evidence, the Grading of Recommendations Assessment, Development and Evaluation (GRADE) approach was used^36^.

### Outcomes of interest

Our primary endpoint was postoperative bile leak, including bile leak and biloma, reported in the studies. Secondary endpoints were postoperative bleeding (i.e., any kind of bleeding related to the operation or the drain insertion, such as drain site bleeding, etc.); interventional percutaneous drainage including any kind of drain fitted postoperatively to treat a symptomatic fluid collection; wound infection (defined as reported wound or drain site infection; total complications including all surgical and non-surgical complications; length of hospital stay; and type of intraabdominal inserted drains.

### Statistical analysis

All data were analyzed by RevMan version 5.3 (Nordic Cochrane Centre, Cochrane Collaboration, Copenhagen, Denmark). The effect size for dichotomous outcomes was measured as odds ratios (OR). For continuous outcomes, weighted mean differences (MDs) were computed by the inverse variance method. Summary effect measures are presented together with their corresponding 95% confidence intervals (CI). Statistical heterogeneity was evaluated with the I^2^ statistic. I^2^ values between 0 and 25% indicate insignificant heterogeneity, 26% and 50% indicate low heterogeneity, 51% and 75% indicate moderate heterogeneity, and 76% and 100% indicate high heterogeneity. The fixed-effects model was used when the I^2^ was < 50%. When the I^2^ was > 50%, the random-effects model was utilized. The methods of Wan et al.^37^ were applied to estimate mean (standard deviation) values for studies that reported only medians and ranges. A p-value less than 0.05 was considered statistically significant in all analyses.

## Supplementary Information

Supplementary Information

## Abbreviations

OR: Odds ratios
MDs: Mean differences
CI: Confidence intervals
RCTs: Randomized controlled trials
PRISMA: Preferred reporting items for systematic reviews and meta-analyses
PICOS: Population, intervention, comparison, outcomes, and design of studies
MINORS: Methodological index for non‐randomized studies
ISGLS: International study group of liver surgery
