# Supplementary material for: Systematic review and meta-analysis of the efficacy of prophylactic abdominal drainage in major liver resections
Source: Sci Rep. 2021 Feb 4;11:3095. doi: 10.1038/s41598-021-82333-x (PMC7862226; doi:10.1038/s41598-021-82333-x)

**Systematic review and Meta-analysis of the Efficacy of Prophylactic Abdominal Drainage in Major Liver Resections**

Sepehr Abbasi Dezfouli^1^, Umut Kaan Ünal^1^, Omid Ghamarnejad^1^, Elias Khajeh^1,^ Sadeq Ali-Hasan-Al-Saegh^1^, Ali Ramouz^1^, Roozbeh Salehpour^1^, Mohammad Golriz^1^, De-Hua Chang^2^, Markus Mieth^1^, Katrin Hoffmann^1,3^, Pascal Probst^1^ and Arianeb Mehrabi^1,3^

^1^Department of General, Visceral, and Transplantation Surgery, University of Heidelberg, Heidelberg, Germany

^2^Department of Diagnostic and Interventional Radiology, University of Heidelberg, Heidelberg, Germany

^3^Liver Cancer Center Heidelberg (LCCH), Heidelberg, Germany

**Short title:** Intraoperative abdominal drainage in major liver resection

**Number of Tables:** 3

**Number of Figures:** 7

**Word count:** 3902

**Correspondence to:**

Prof. Dr. med. A. Mehrabi, FICS, FEBS, FACS

Head of the Division of Liver Surgery and Visceral Transplantation

Department of General, Visceral, and Transplantation Surgery

University of Heidelberg

Im Neuenheimer Feld 110

69120 Heidelberg, Germany

Tel: 0049 – 6221 – 5636223; Fax: 0049 – 6221 - 567470

E-Mail: arianeb.mehrabi@med.uni-heidelberg.de

**Conflicts of Interest:** None

**Funding:** None

**Supplementary figures:**

**Supp. figure 1.** Forest plot of postoperative bile leak in randomized controlled trials.


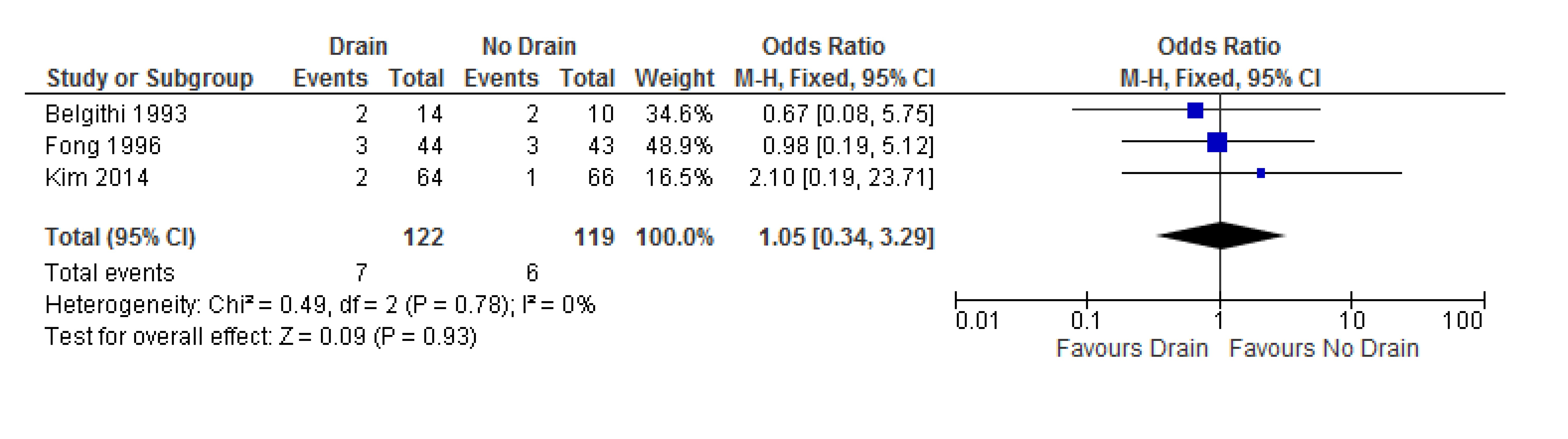


**Supp. figure 2.** Forest plot of postoperative bile leak in non-randomized studies.


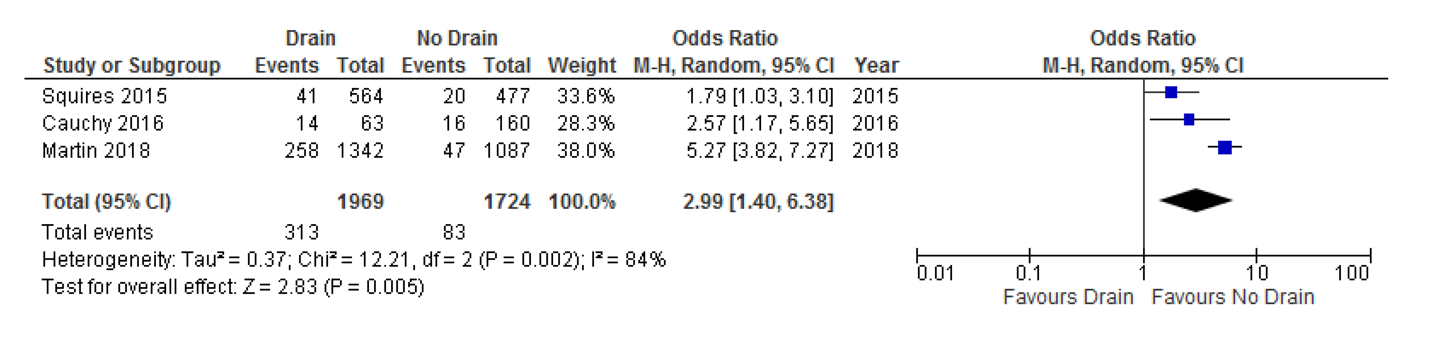

Supplement: Supplementary file 1 — Supplementary Information [file 41598_2021_82333_MOESM1_ESM.docx]
